# Establishing a nomogram to predict the risk of pulmonary embolism in tumor wards: A retrospective study

**DOI:** 10.1097/MD.0000000000045737

**Published:** 2025-11-21

**Authors:** Qiu Liuyi, Chen Tenggao, Lu Yifang, Li Wenchen, Chen Jianping, Ma Xu

**Affiliations:** a Department of Pathology, Affiliated Dongyang Hospital of Wenzhou Medical University, Dongyang, Zhejiang Province, China; b Department of Oncology Surgery, Affiliated Dongyang Hospital of Wenzhou Medical University, Dongyang, Zhejiang Province, China; c Department of Internal Medicine-Oncology, Affiliated Dongyang Hospital of Wenzhou Medical University, Dongyang, Zhejiang Province, China; d Department of Neurology, Affiliated Dongyang Hospital of Wenzhou Medical University, Dongyang, Zhejiang Province, China; e Department of Emergency, Affiliated Dongyang Hospital of Wenzhou Medical University, Dongyang, Zhejiang Province, China; f Department of Vascular Surgery, Affiliated Dongyang Hospital of Wenzhou Medical University, Dongyang, Zhejiang Province, China.

**Keywords:** clinical symptoms, oncology, prediction model, pulmonary embolism, retrospective analysis

## Abstract

Pulmonary embolism (PE) is a life-threatening disease with high morbidity and mortality in patients with cancer. To enhance medical treatment and management, this study aimed to create a nomogram to accurately predict PE risk in patients in tumor wards. In this retrospective study, we obtained information on medical history, complications, clinical characteristics, and laboratory biomarkers from patients with suspected PE admitted to the Oncology Department at the Affiliated Dongyang Hospital of Wenzhou Medical University between January 2012 and December 2021. A total of 512 patients were randomly divided into training and validation groups at a 6:4 ratio. A nomogram-based scoring model was developed using least absolute shrinkage and selection operator and multivariate logistic regressions, and its performance was evaluated using receiver operating characteristic, calibration, and clinical decision curves. Analysis of over 50 features from the patients led to a model based on 5 predictive features: neutrophil count, sex, systolic blood pressure, surgical status, and D-dimer levels. The model achieved area under the receiver operating characteristic curve values of 0.758 and 0.702 in the training and validation cohorts, respectively. It demonstrated a sensitivity of 85.58%, a specificity of 35.78%, a positive predictive value of 72.44%, and a negative predictive value of 55.71%. The calibration curve showed strong consistency between the predicted and actual probabilities, and decision curve analysis confirmed a favorable net clinical benefit. In conclusion, we successfully developed a novel numerical model that can predict PE risk in oncology patients, enabling the appropriate selection of prevention strategies and helping to reduce unnecessary computed tomography pulmonary angiography scans and their associated adverse effects.

## 1. Introduction

Oncology patients are susceptible to pulmonary embolism (PE), a life-threatening condition caused by blockage of pulmonary arteries or branches by various emboli.^[1]^ Diagnosing PE is challenging owing to its nonspecific symptoms,^[2]^ and the high misdiagnosis and mortality rates make PE an important medical issue. The currently recommended diagnostic method, computed tomography pulmonary angiography (CTPA),^[3]^ is an expensive and time-consuming procedure that can also cause serious side effects in patients.

The development of predictive models for PE in tumor wards has received increasing attention. Numerous studies have been conducted to develop and validate models for predicting PE risk in this population.^[4–9]^ In previous studies,^[10,11]^ prediction models were developed using clinical and biological variables in oncology patients. They showed that age, immobility, previous thromboembolism events, and the presence of cancer were independent risk factors for PE in this population. Previous studies^[12]^ have used a combination of demographic, clinical, and laboratory parameters to create a predictive model for PE. Elevated D-dimer levels and the presence of deep vein thrombosis are significant predictors of PE in oncology patients. Other studies^[13,14]^ have evaluated the clinical utility of a simplified Geneva score in predicting PE, which could serve as a quick and easy screening tool for healthcare professionals. In a more recent study,^[15–19]^ the authors used machine learning algorithms to develop a predictive model for PE; the model had high accuracy in predicting PE and outperformed traditional risk assessment tools, such as the Wells score. They collected data from the electronic medical records of inpatients with and without PE and used machine learning algorithms to develop a risk-prediction model.^[17]^ In practice, evaluating the risk of PE using machine learning algorithms is still rare, possibly because machine learning algorithms require the cooperation of engineers and clinical doctors, which poses obstacles to practical application. These models have the potential to improve the diagnosis and management of PE in medical and surgical inpatients. The clinical manifestations of PE are nonspecific, and there are significant differences among different disease groups. However, there are currently few predictive models for PE in tumor wards; therefore, a simple and rapid risk prediction model is essential.

This study aimed to develop and validate a numerical model using electronic medical record data from patients in our tumor ward to predict PE.

## 2. Materials and methods

### 2.1. Ethics statement

This retrospective study was approved by the affiliated Dongyang Hospital of Wenzhou Medical University (Dongyang, China) Institutional Review Board (approval no. 2022-YX-160), and the requirement for informed consent was waived. Patient information was kept confidential, and all identifying information was deleted prior to evaluation. This study adhered to the tenets of the Declaration of Helsinki.

### 2.2. Study participants

Patients with suspected PE admitted to the tumor ward of our hospital between January 2012 and December 2021 were enrolled in this study. To ensure reliability, we excluded patients with unclear CTPA diagnostic reports. The Clinical Research Data Platform was used to retrospectively collect patient data. The baseline data were screened and extracted. The medical records of 512 patients were statistically analyzed. The common proportions between the training and validation groups were 5:5, 6:4, 7:3, and 8:2, with 6:4 and 7:3 being the most common. The total number of cases in this study was small (n = 512). Thus, to achieve a better balance between the 2 groups, we used a 6:4 ratio.

### 2.3. Data collection and observation indicators

**Figure 1.**
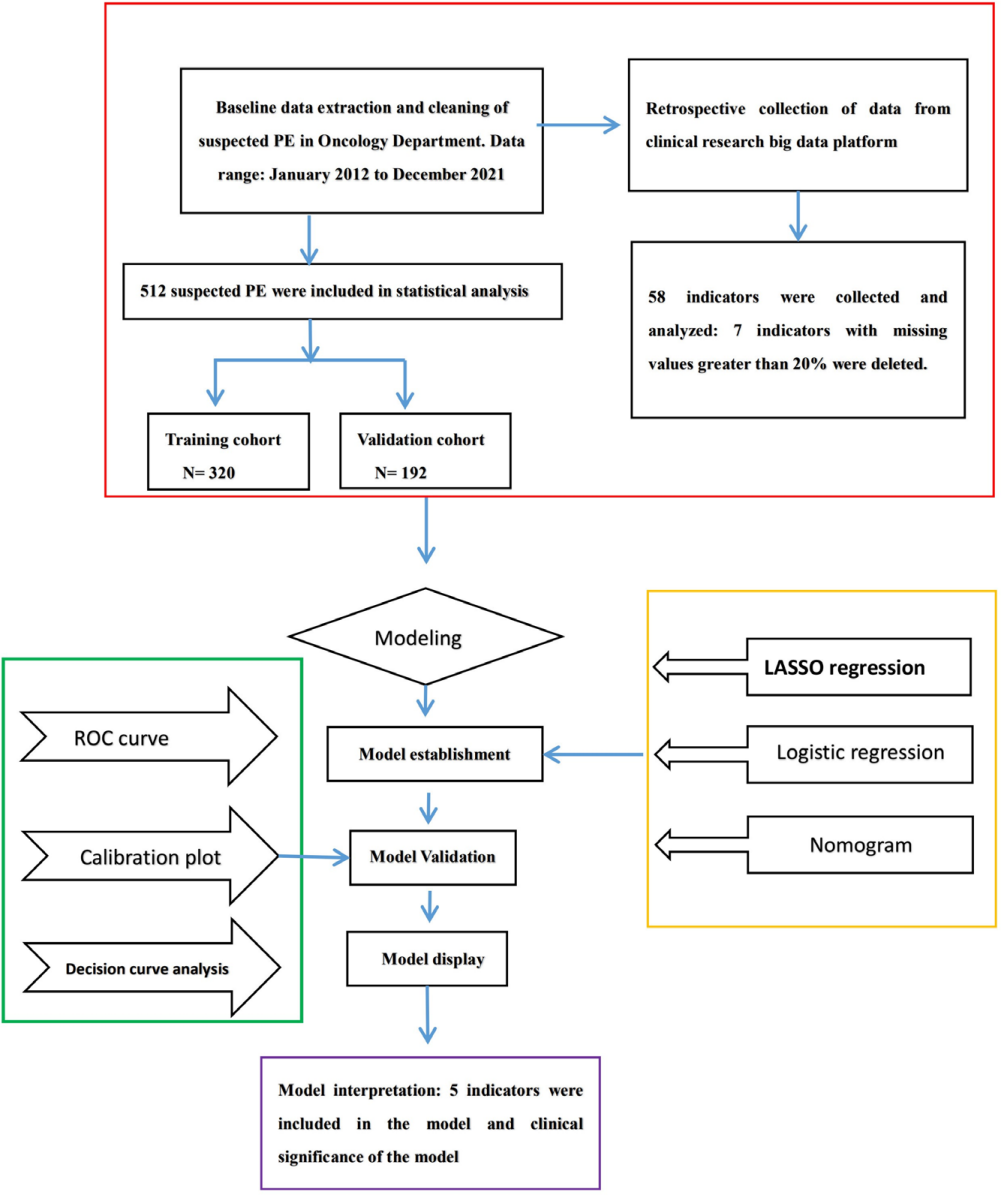
Flowchart of the PE prediction step. PE = pulmonary embolism.

PE was defined according to the criteria of the European Society of Cardiology guidelines.^[20]^ CTPA was used to confirm PE, including subsegmental PE, by identifying filling defects in the pulmonary arterial system. Patients who undergo CTPA are suspected to have PE. Using a strict definition of indicators, we collected data on medical history, complications, individual clinical characteristics, and clinical biomarkers. For example, the lowest values were selected for blood oxygen saturation and systolic and diastolic blood pressure from admission to CTPA, whereas the highest values were selected for other indicators. Figure 1 presents the research flowchart. Considering that this study used a diagnostic predictive model, the Tripod Checklist was selected to ensure compliance with publication standards.

### 2.4. Statistical analysis

R Studio software for Windows was used for the data analysis. Student *t* test or Mann–Whitney *U* test was used to compare continuous variables, expressed as the mean and standard deviation or median and interquartile range. Categorical variables are expressed as frequencies and percentages, and the χ^2^ test or Fisher exact test was used for comparison. Data were available for 58 variables for all the participants. Among the variables, 7 indicators with more than 20% missing values were deleted to ensure data reliability. To impute the remaining missing predictor values, multiple imputation techniques were performed using the “mice” package.^[21]^ To select the optimal predictive features, the Least Absolute Shrinkage and Selection Operator (LASSO) regression analysis was used with the “glmnet” package.^[22]^ A numerical model was constructed using multivariate logistic regression analysis with the “rms” package. The “regplot” package was used to construct a nomogram. The “reportROC” package was used to calculate the performance of the diagnostic indicators for the model. The results are expressed as odds ratios (ORs) with 95% confidence intervals (CIs). Statistical significance was determined using 2-sided *P* < .05.

### 2.5. Model development, validation, and evaluation

Multivariable logistic regression analysis was performed to develop a PE prediction model by combining the selected predictors from LASSO analysis. The performance of the models was assessed using various evaluation metrics, including area under the receiver operating characteristic (ROC) curve (AUC), calibration curves, and decision curve analysis (DCA).^[23]^ AUC was used to measure model discrimination, while a calibration curve was used to assess the consistency between the predicted and actual probabilities of PE. DCA was used to quantify net clinical benefits at different thresholds.

## 3. Results

### 3.1. Study population characteristics

Due to an excessive amount of missing data (≤20%), 7 variables were removed from our data before proceeding with the analysis. The present study included 51 variables with < 20% missing data (see Figure S1, Supplemental Digital Content, https://links.lww.com/MD/Q710, which shows the variables with < 20% missing data). The percentage of missing data was 0.00 − 19.34% for these variables. For an incidence rate of 31.25%, 512 participants with suspected PE were included. Table 1 shows the demographic and clinical characteristics of the patients. Table 2 summarizes the characteristics of the randomized participants in the 2 groups: training (n = 320) and validation (n = 192) cohorts.

**Table 1:** Baseline characteristics of the subjects.

Variables	Total (n = 512)	No PE (n = 352)	PE (n = 160)	*P*
Sex, n (%)				.514
Female	214 (41.8)	151 (42.9)	63 (39.4)	
Male	298 (58.2)	201 (57.1)	97 (60.6)	
Age (yr), Median (Q1, Q3)	68 (60.75, 75)	67 (60, 74)	69 (63, 76)	.035
Temperature (°C), Median (Q1, Q3)	37.8 (37, 38.5)	37.8 (37, 38.5)	37.8 (37, 38.4)	.87
Breathing (breaths/min), Median (Q1, Q3)	22 (20, 26)	22 (20, 26)	23.5 (22, 26)	.005
Pulse (beats/min), Median (Q1, Q3)	105 (94, 120)	104 (92, 120)	106.5 (97, 120)	.17
Systolic blood pressure (mm Hg), Median (Q1, Q3)	96 (89, 109)	100 (90, 112)	91 (87, 100)	<.001
Diastolic pressure (mm Hg), Median (Q1, Q3)	55 (48, 63)	57 (50, 65.25)	52 (47, 59)	<.001
Headache, n (%)				1
No	505 (98.6)	347 (98.6)	158 (98.8)	
Yes	7 (1.4)	5 (1.4)	2 (1.2)	
Dizziness, n (%)				.565
No	498 (97.3)	341 (96.9)	157 (98.1)	
Yes	14 (2.7)	11 (3.1)	3 (1.9)	
Chest tightness, n (%)				.257
No	450 (87.9)	305 (86.6)	145 (90.6)	
Yes	62 (12.1)	47 (13.4)	15 (9.4)	
Anhelation, n (%)				.388
No	467 (91.2)	318 (90.3)	149 (93.1)	
Yes	45 (8.8)	34 (9.7)	11 (6.9)	
Hemoptysis, n (%)				.71
No	504 (98.4)	347 (98.6)	157 (98.1)	
Yes	8 (1.6)	5 (1.4)	3 (1.9)	
Chest pain, n (%)				1
No	504 (98.4)	346 (98.3)	158 (98.8)	
Yes	8 (1.6)	6 (1.7)	2 (1.2)	
Syncope, n (%)				.65
No	507 (99)	349 (99.1)	158 (98.8)	
Yes	5 (1)	3 (0.9)	2 (1.2)	
Cough, n (%)				.291
No	420 (82)	284 (80.7)	136 (85)	
Yes	92 (18)	68 (19.3)	24 (15)	
Fever, n (%)				.357
No	500 (97.7)	342 (97.2)	158 (98.8)	
Yes	12 (2.3)	10 (2.8)	2 (1.2)	
Lower limb edema, n (%)				1
No	503 (98.2)	346 (98.3)	157 (98.1)	
Yes	9 (1.8)	6 (1.7)	3 (1.9)	
COPD, n (%)				.038
No	453 (88.5)	304 (86.4)	149 (93.1)	
Yes	59 (11.5)	48 (13.6)	11 (6.9)	
Hypertension, n (%)				.873
No	319 (62.3)	218 (61.9)	101 (63.1)	
Yes	193 (37.7)	134 (38.1)	59 (36.9)	
Diabetes, n (%)				1
No	467 (91.2)	321 (91.2)	146 (91.2)	
Yes	45 (8.8)	31 (8.8)	14 (8.8)	
Coronary heart disease, n (%)				.461
No	458 (89.5)	312 (88.6)	146 (91.2)	
Yes	54 (10.5)	40 (11.4)	14 (8.8)	
Hyperlipidemia, n (%)				.105
No	505 (98.6)	345 (98)	160 (100)	
Yes	7 (1.4)	7 (2)	0 (0)	
Atrial fibrillation, n (%)				.86
No	490 (95.7)	336 (95.5)	154 (96.2)	
Yes	22 (4.3)	16 (4.5)	6 (3.8)	
Surgery, n (%)				<.001
No	384 (75)	287 (81.5)	97 (60.6)	
Yes	128 (25)	65 (18.5)	63 (39.4)	
Tumor, n (%)				.551
No	109 (21.3)	78 (22.2)	31 (19.4)	
Yes	403 (78.7)	274 (77.8)	129 (80.6)	
Smoking, n (%)				.675
No	322 (62.9)	224 (63.6)	98 (61.3)	
Yes	190 (37.1)	128 (36.4)	62 (38.8)	
Drinking, n (%)				1
No	339 (66.2)	233 (66.2)	106 (66.2)	
Yes	173 (33.8)	119 (33.8)	54 (33.8)	
WBC (10^9^/L), Median (Q1, Q3)	9.18 (6.46, 12.89)	8.81 (6.14, 12.83)	10.17 (7.28, 13.14)	.025
RBC (10^12^/L), Median (Q1, Q3)	4.19 (3.72, 4.6)	4.2 (3.71, 4.58)	4.18 (3.77, 4.64)	.722
Mg (mmol/L), Median (Q1, Q3)	0.9 (0.84, 0.95)	0.9 (0.84, 0.94)	0.89 (0.84, 0.95)	.831
HGB (g/L), Median (Q1, Q3)	124 (108, 138)	124 (107, 137)	126 (109, 140.25)	.302
Hct, Median (Q1, Q3)	0.38 (0.33, 0.42)	0.37 (0.33, 0.41)	0.38 (0.34, 0.42)	.279
Neutrophil percent, Median (Q1, Q3)	0.85 (0.75, 0.9)	0.83 (0.73, 0.89)	0.86 (0.81, 0.91)	<.001
Neutrophil count (10^9^/L), Median (Q1, Q3)	7.35 (4.57, 10.79)	6.54 (4.25, 10.76)	8.37 (5.45, 11.04)	.003
Lymphocyte Percent, Median (Q1, Q3)	0.25 (0.19, 0.33)	0.25 (0.18, 0.34)	0.25 (0.19, 0.33)	.573
Lymphocyte count (10^9^/L), Median (Q1, Q3)	1.41 (1.04, 1.9)	1.41 (1, 1.89)	1.41 (1.1, 2.01)	.276
PLT (10^9^/L), Median (Q1, Q3)	233 (176, 298.25)	231 (173.75, 290.25)	240 (181.5, 306.25)	.48
ALB (g/L), Median (Q1, Q3)	38.5 (33.98, 41.7)	38.6 (34.07, 41.43)	38.5 (33.57, 42.15)	.608
PDW (%), Median (Q1, Q3)	15.3 (11.9, 16.3)	15.3 (11.9, 16.3)	15.5 (12.08, 16.3)	.254
RDW (%), Median (Q1, Q3)	0.14 (0.13, 0.16)	0.14 (0.13, 0.16)	0.14 (0.13, 0.16)	.783
HDL (mmol/L), Median (Q1, Q3)	1.05 (0.87, 1.29)	1.05 (0.88, 1.28)	1.06 (0.86, 1.33)	.725
LDL (mmol/L), Median (Q1, Q3)	2.69 (2.11, 3.25)	2.67 (2.11, 3.25)	2.72 (2.13, 3.21)	.552
Apolipoprotein A1 (g/L), Median (Q1, Q3)	1.08 (0.88, 1.35)	1.08 (0.87, 1.32)	1.11 (0.89, 1.42)	.295
Apolipoprotein B (g/L), Median (Q1, Q3)	0.93 (0.78, 1.14)	0.94 (0.78, 1.14)	0.93 (0.8, 1.17)	.704
TGs (mmol/L), Median (Q1, Q3)	1.43 (1.11, 1.98)	1.44 (1.11, 1.97)	1.41 (1.13, 2.02)	.7
TC (mmol/L), Median (Q1, Q3)	4.42 (3.74, 5.12)	4.42 (3.74, 5.07)	4.42 (3.75, 5.21)	.55
Fibrinogen (g/L), Median (Q1, Q3)	4.47 (3.55, 5.74)	4.51 (3.54, 5.79)	4.36 (3.56, 5.34)	.175
D-dimer (mg/L), Median (Q1, Q3)	6.48 (2.09, 13.04)	4.51 (1.5, 10.32)	10.64 (6.57, 16)	<.001
PT (s), Median (Q1, Q3)	14.1 (13.3, 15.2)	14 (13.3, 15.1)	14.3 (13.4, 15.2)	.169
APTT (s), Median (Q1, Q3)	39.7 (36, 44.3)	39.85 (36.2, 44.4)	39.25 (35.77, 43.8)	.352
TT (s), Median (Q1, Q3)	16.1 (15.5, 16.9)	16.2 (15.5, 16.9)	16.1 (15.6, 16.9)	.8

ALB = albumin, APTT = Activated partial prothrombin time, Hct = Hematocrit, HDL = High-density lipoprotein, HGB = hemoglobin, LDL = low-density lipoprotein, Mg = magnesium, PDW = Platelet distribution width, PLT = platelet, PT = prothrombin time, RBC = Red blood cell, RDW = Red blood cell distribution width, TC = total cholesterol, TGs = triglycerides, TT = Thrombin time, WBC = White blood cell.

**Table 2:** The baseline characteristics of the patients included in the training and validation cohorts.

Variables	Total (n = 512)	Validation (n = 192)	Training (n = 320)	*P*
PE, n (%)				.922
No	352 (68.8)	131 (68.2)	221 (69.1)	
Yes	160 (31.2)	61 (31.8)	99 (30.9)	
Sex, n (%)				.432
Female	214 (41.8)	85 (44.3)	129 (40.3)	
Male	298 (58.2)	107 (55.7)	191 (59.7)	
Age (years), Median (Q1, Q3),	68 (60.75, 75)	68 (59, 76)	68 (62, 74)	.859
Temperature (°C), Median (Q1, Q3)	37.8 (37, 38.5)	37.8 (37.18, 38.5)	37.8 (37, 38.5)	.545
Breathing (breaths/min), Median (Q1, Q3)	22 (20, 26)	22 (20, 26)	22 (20, 27)	.259
Pulse (beats/min), Median (Q1, Q3)	105 (94, 120)	106 (96, 120)	104 (94, 120)	.453
Systolic blood pressure (mm Hg), Median (Q1, Q3)	96 (89, 109)	96.5 (90, 109)	95 (89, 109)	.471
Diastolic blood pressure (mm Hg), Median (Q1, Q3)	55 (48, 63)	54 (48.75, 64)	55 (47.75, 62.25)	.749
Headache, n (%)				.049
No	505 (98.6)	192 (100)	313 (97.8)	
Yes	7 (1.4)	0 (0)	7 (2.2)	
Dizziness, n (%)				.124
No	498 (97.3)	190 (99)	308 (96.2)	
Yes	14 (2.7)	2 (1)	12 (3.8)	
Chest tightness, n (%)				.624
No	450 (87.9)	171 (89.1)	279 (87.2)	
Yes	62 (12.1)	21 (10.9)	41 (12.8)	
Anhelation, n (%)				.904
No	467 (91.2)	176 (91.7)	291 (90.9)	
Yes	45 (8.8)	16 (8.3)	29 (9.1)	
Hemoptysis, n (%)				.716
No	504 (98.4)	190 (99)	314 (98.1)	
Yes	8 (1.6)	2 (1)	6 (1.9)	
Chest pain, n (%)				.481
No	504 (98.4)	188 (97.9)	316 (98.8)	
Yes	8 (1.6)	4 (2.1)	4 (1.2)	
Syncope, n (%)				.068
No	507 (99)	188 (97.9)	319 (99.7)	
Yes	5 (1)	4 (2.1)	1 (0.3)	
Cough, n (%)				.812
No	420 (82)	156 (81.2)	264 (82.5)	
Yes	92 (18)	36 (18.8)	56 (17.5)	
Fever, n (%)				1
No	500 (97.7)	188 (97.9)	312 (97.5)	
Yes	12 (2.3)	4 (2.1)	8 (2.5)	
Lower limb edema, n (%)				1
No	503 (98.2)	189 (98.4)	314 (98.1)	
Yes	9 (1.8)	3 (1.6)	6 (1.9)	
COPD, n (%)				.453
No	453 (88.5)	173 (90.1)	280 (87.5)	
Yes	59 (11.5)	19 (9.9)	40 (12.5)	
Hypertension, n (%)				.981
No	319 (62.3)	119 (62)	200 (62.5)	
Yes	193 (37.7)	73 (38)	120 (37.5)	
Diabetes, n (%)				.658
No	467 (91.2)	177 (92.2)	290 (90.6)	
Yes	45 (8.8)	15 (7.8)	30 (9.4)	
Coronary heart disease, n (%)				.414
No	458 (89.5)	175 (91.1)	283 (88.4)	
Yes	54 (10.5)	17 (8.9)	37 (11.6)	
Hyperlipidemia, n (%)				1
No	505 (98.6)	189 (98.4)	316 (98.8)	
Yes	7 (1.4)	3 (1.6)	4 (1.2)	
Atrial fibrillation, n (%)				.574
No	490 (95.7)	182 (94.8)	308 (96.2)	
Yes	22 (4.3)	10 (5.2)	12 (3.8)	
Surgery, n (%)				.343
No	384 (75)	139 (72.4)	245 (76.6)	
Yes	128 (25)	53 (27.6)	75 (23.4)	
Tumor, n (%)				.596
No	109 (21.3)	38 (19.8)	71 (22.2)	
Yes	403 (78.7)	154 (80.2)	249 (77.8)	
Smoking, n (%)				.813
No	322 (62.9)	119 (62)	203 (63.4)	
Yes	190 (37.1)	73 (38)	117 (36.6)	
Drinking, n (%)				.904
No	339 (66.2)	126 (65.6)	213 (66.6)	
Yes	173 (33.8)	66 (34.4)	107 (33.4)	
WBC (10^9^/L), Median (Q1, Q3)	9.18 (6.46, 12.89)	9.09 (6.55, 13.44)	9.22 (6.41, 12.32)	.319
RBC (10^12^/L), Median (Q1, Q3)	4.19 (3.72, 4.6)	4.26 (3.71, 4.7)	4.18 (3.73, 4.55)	.302
Mg (mmol/L), Median (Q1, Q3)	0.9 (0.84, 0.95)	0.89 (0.85, 0.94)	0.9 (0.84, 0.95)	.56
HGB (g/L), Median (Q1, Q3)	124 (108, 138)	124 (107.75, 138)	124 (108, 137.25)	.693
Hct, Median (Q1, Q3)	0.38 (0.33, 0.42)	0.38 (0.33, 0.42)	0.37 (0.33, 0.42)	.44
Neutrophil Percent, Median (Q1, Q3)	0.85 (0.75, 0.9)	0.86 (0.76, 0.9)	0.84 (0.73, 0.9)	.061
Neutrophil count (10^9^/L), Median (Q1, Q3)	7.35 (4.57, 10.79)	7.63 (4.74, 11.87)	7.14 (4.35, 10.44)	.117
Lymphocyte Percent, Median (Q1, Q3)	0.25 (0.19, 0.33)	0.26 (0.18, 0.34)	0.25 (0.19, 0.33)	.547
Lymphocyte count (10^9^/L), Median (Q1, Q3)	1.41 (1.04, 1.9)	1.44 (1.1, 1.95)	1.41 (1.02, 1.89)	.332
PLT (10^9^/L), Median (Q1, Q3)	233 (176, 298.25)	248 (181, 312)	228 (173, 288.25)	.026
ALB (g/L), Median (Q1, Q3)	38.5 (33.98, 41.7)	38.9 (35, 41.93)	38.2 (33.6, 41.6)	.066
PDW (%), Median (Q1, Q3)	15.3 (11.9, 16.3)	15.5 (11.9, 16.4)	15.2 (11.97, 16.23)	.424
RDW (%), Median (Q1, Q3)	0.14 (0.13, 0.16)	0.14 (0.13, 0.16)	0.14 (0.13, 0.16)	.434
HDL (mmol/L), Median (Q1, Q3)	1.05 (0.87, 1.29)	1.05 (0.89, 1.28)	1.05 (0.85, 1.3)	.269
LDL (mmol/L), Median (Q1, Q3)	2.69 (2.11, 3.25)	2.84 (2.29, 3.3)	2.61 (2.03, 3.16)	.006
Apolipoprotein A1 (g/L), Median (Q1, Q3)	1.08 (0.88, 1.35)	1.12 (0.92, 1.39)	1.07 (0.85, 1.33)	.072
Apolipoprotein B (g/L), Median (Q1, Q3)	0.93 (0.78, 1.14)	0.97 (0.81, 1.21)	0.91 (0.76, 1.1)	.012
TGs (mmol/L), Median (Q1, Q3)	1.43 (1.11, 1.98)	1.47 (1.15, 1.89)	1.4 (1.1, 1.99)	.533
TC (mmol/L), Median (Q1, Q3)	4.42 (3.74, 5.12)	4.62 (3.9, 5.28)	4.32 (3.67, 5.01)	.006
Fibrinogen (g/L), Median (Q1, Q3)	4.47 (3.55, 5.74)	4.52 (3.63, 5.6)	4.45 (3.52, 5.76)	.384
D-dimer (mg/L), Median (Q1, Q3)	6.48 (2.09, 13.04)	6.28 (2.02, 12.59)	6.93 (2.13, 13.66)	.793
PT (s), Median (Q1, Q3)	14.1 (13.3, 15.2)	13.95 (13.2, 15)	14.2 (13.37, 15.2)	.037
APTT (s), Median (Q1, Q3)	39.7 (36, 44.3)	39.05 (35.48, 44.1)	40.1 (36.3, 44.3)	.039
TT (s), Median (Q1, Q3)	16.1 (15.5, 16.9)	16.2 (15.5, 16.82)	16.1 (15.58, 17)	.727

ALB = albumin, APTT = Activated partial prothrombin time, Hct = Hematocrit, HDL = High-density lipoprotein, HGB = hemoglobin, LDL = low-density lipoprotein, Mg = magnesium, PDW = Platelet distribution width, PLT = platelet, PT = prothrombin time, RBC = Red blood cell, RDW = Red blood cell distribution width, TC = total cholesterol, TGs = triglycerides, TT = Thrombin time, WBC = White blood cell.

### 3.2. Selected predictors

**Figure 2.**
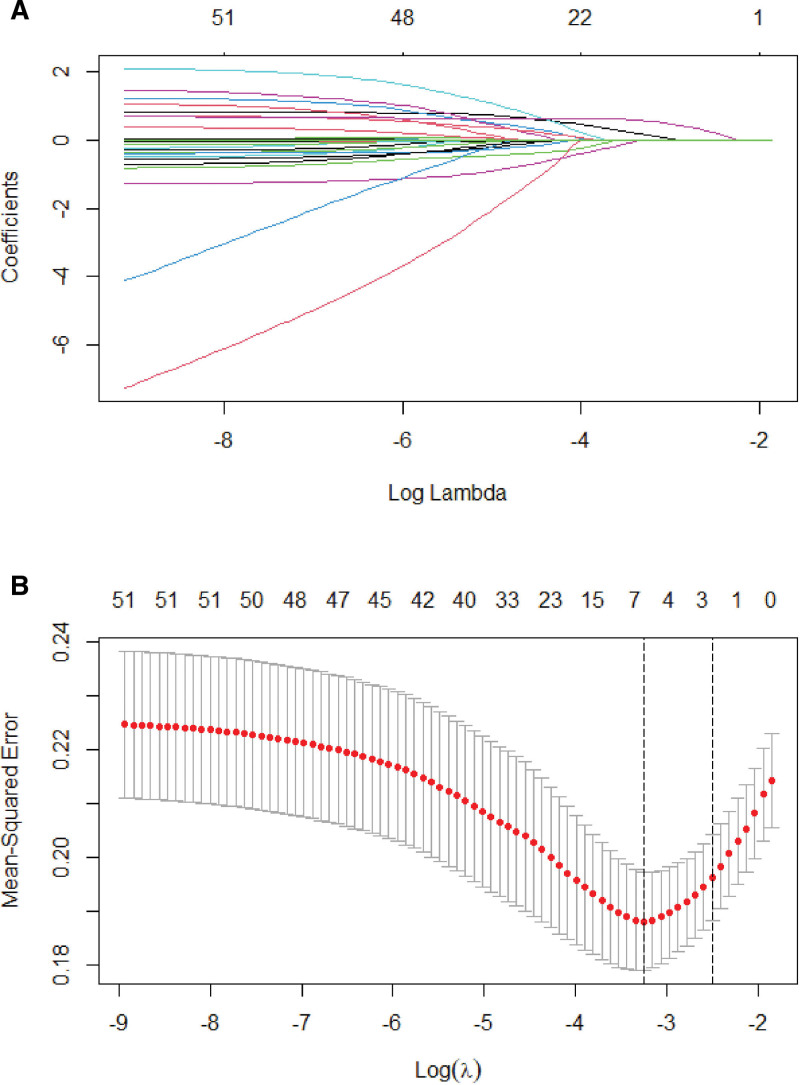
LASSO regression tuning parameter selection in the training cohort. (A) LASSO coefficients of the 51 clinical characteristics, plotted against log (lambda). A vertical line was drawn at the value selected by 10-fold cross-validation. Optimal lambda yielded 5 nonzero coefficient candidates. (B) 10-fold cross-validation over 1-fold mean square error was used to select the optimal candidate (lambda) in the LASSO model. Using the minimum mean square error and 1 SE of the minimum mean square error, dotted vertical lines were drawn at the optimal values. LASSO = least absolute shrinkage and selection operator.

Five potential predictors were ultimately selected from the 51 variables using the LASSO regression analysis (Figure 2A, B). Neutrophil count, sex, systolic blood pressure, surgical status, and D-dimer level were included as optimal predictors. The final prediction model was constructed by multivariable logistic regression analysis using 5 predictors selected from the LASSO regression analysis (Table 3). For our model, the sensitivity was 85.58%, specificity 35.78%, positive prediction value 72.44%, and negative prediction value 55.71%.

**Table 3:** Final model coefficients.

Variables	β	SE	OR	95% CI	*P*
Sex (male/female)	0.607	0.279	1.83	1.06–3.17	.03
Systolic blood pressure (mm Hg)	−0.021	0.008	0.98	0.96–0.99	.01
Surgical status (yes or no)	1.021	0.305	2.77	1.53–5.04	.001
D-dimer levels (mg/L)	0.105	0.022	1.11	1.06–1.16	<.001
Neutrophil count (10^9^/L)	0.032	0.03	1.03	0.97–1.1	.284

### 3.3. Modeling and validation

**Figure 3.**
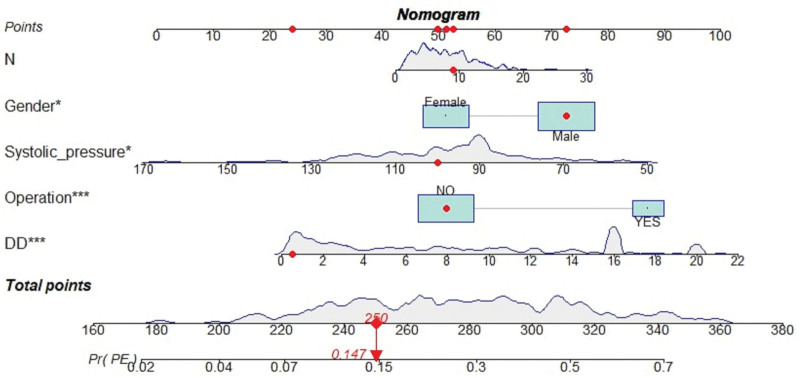
A nomogram based on the combination of 5 indicators obtained using logistic regression analysis. The nomogram was developed in the training dataset with neutrophil count, sex, systolic blood pressure, surgical status, and D-dimer levels. Scores of each feature were added to obtain total scores, and a vertical line was drawn on total scores to obtain the probability of PE. If a patient has a total score of 250, then their probability of developing PE is 0.147 (red numbers). DD = D-dimer, N = neutrophil count.

**Figure 4.**
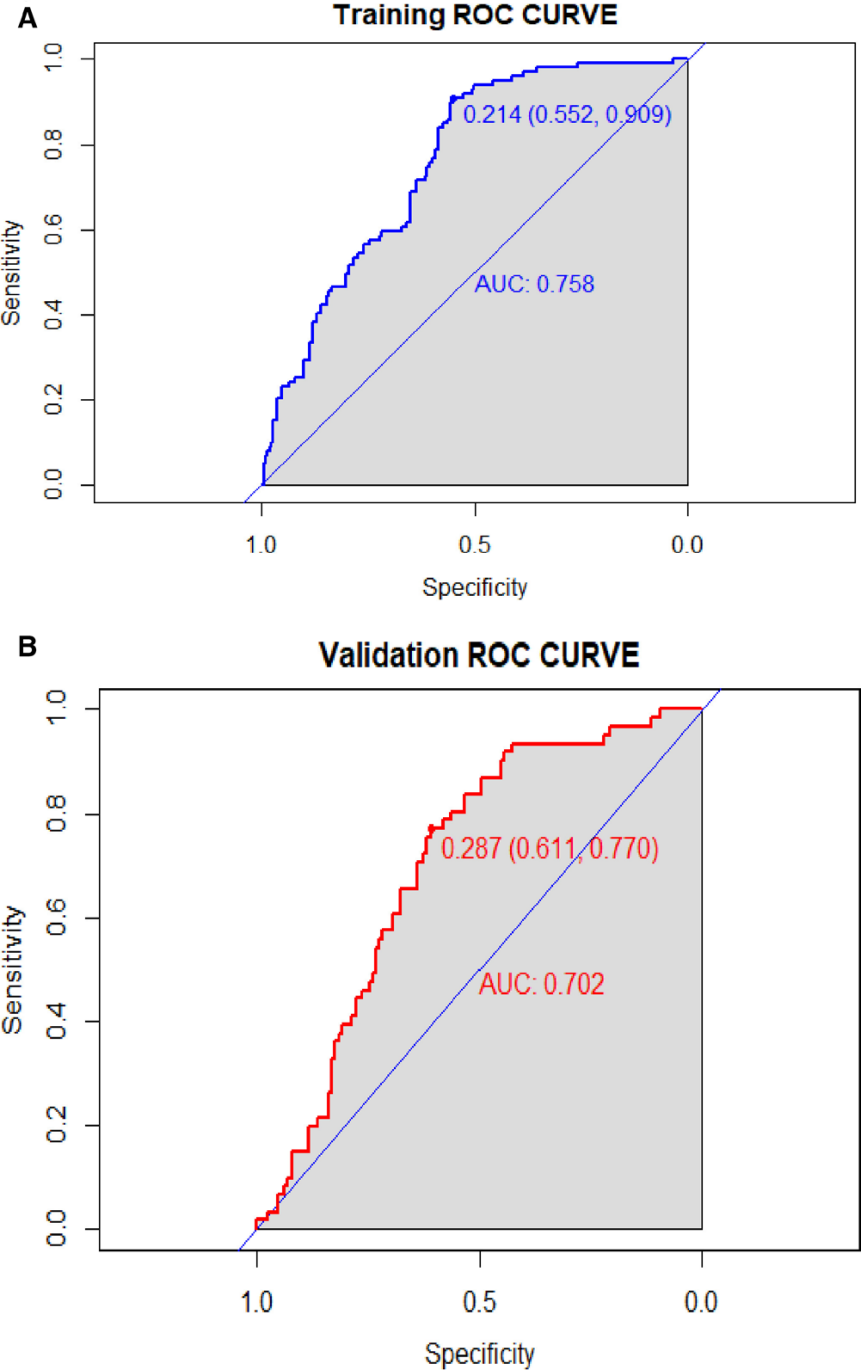
Receiver operating characteristic curves of PE/non-PE discriminating model in training (A) and validation (B) cohorts. ROC curves of the training (AUC-ROC is 0.758) and validation cohorts (AUC-ROC is 0.702). AUC = area under the ROC curve, PE = pulmonary embolism, ROC = receiver operating characteristic.

**Figure 5.**
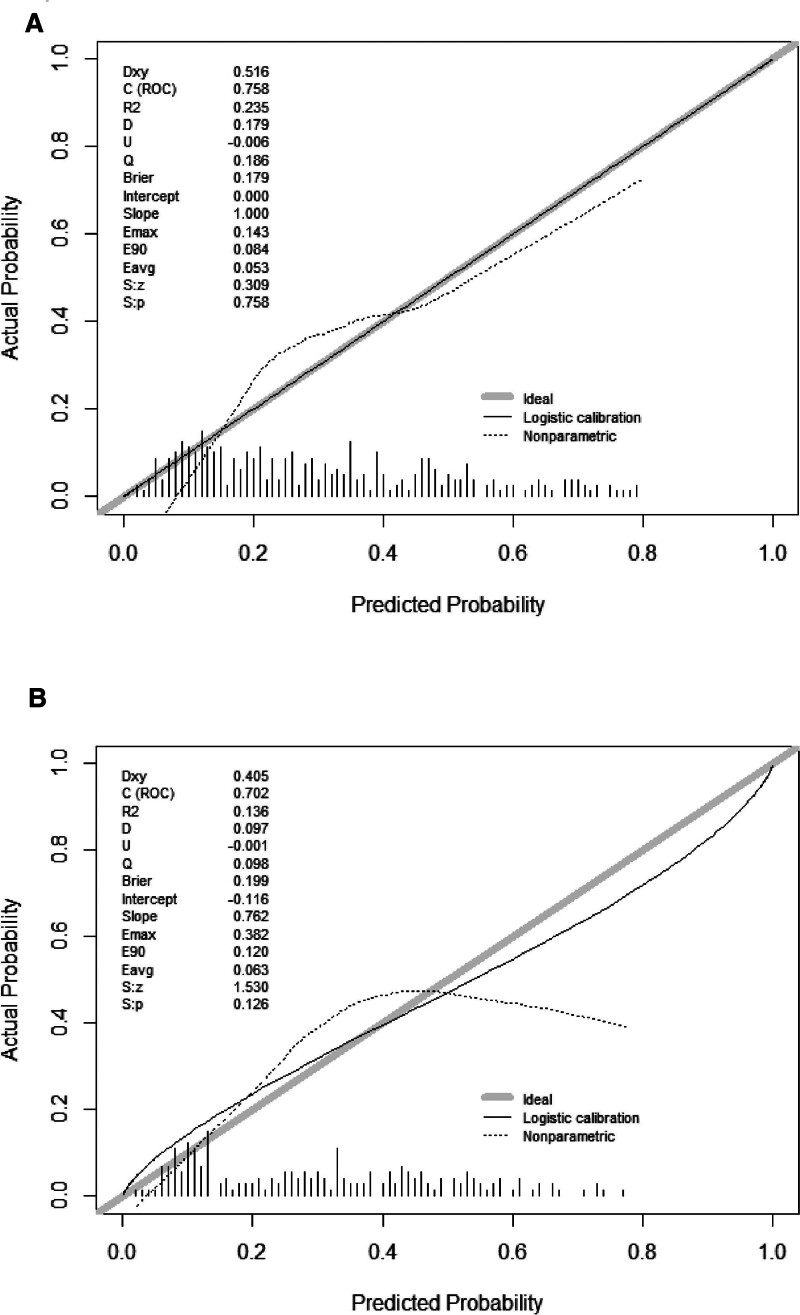
Training (A) and validation (B) cohort calibration curves of the model. The x-axis is the prediction probability of PE. Actual diagnosed PE is on the y-axis. Perfect prediction by an ideal model is represented by the diagonal dashed line. The solid line represents nomogram performance, where a closer fit to the diagonal dotted line indicates a better prognosis. PE = pulmonary embolism.

**Figure 6.**
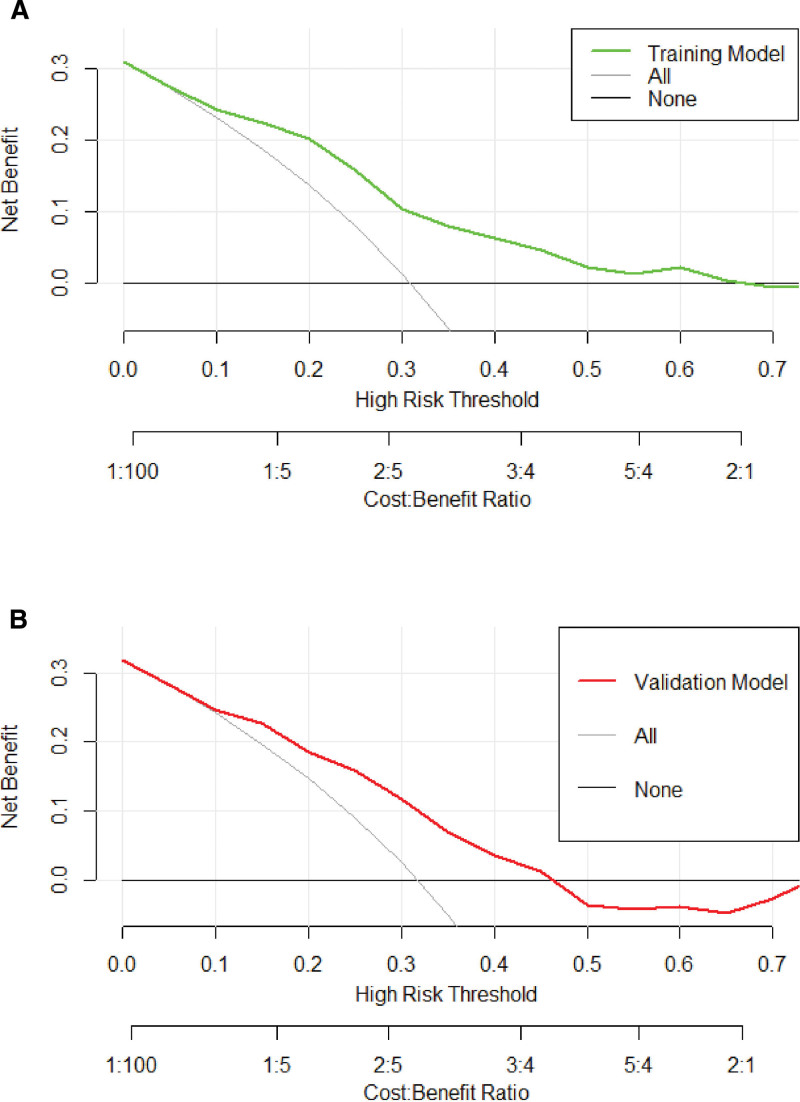
Training (A) and validation (B) cohort decision curve of the model. The black line represents the net benefit when none of the participants are considered to have PE, while the light gray line represents the net benefit when all participants are considered to have PE. The clinical utility of the model is indicated by the area between the no treatment line (black line) and all treatment lines (light gray line) in the model curve. When a risk threshold is <67%, the nomogram model yields a higher net benefit than all treatments (assuming all patients have PE) or no treatments (assuming all patients do not have PE).

We visualized the PE prediction model, as shown in Figure 3. Model discrimination, measured by AUC, was 0.758 (95% CI 0.695 − 0.804) in the training cohort and 0.702 (95% CI 0.630 − 0.776) in the validation cohort. This indicated that the predictive model could effectively discriminate between patients with and without PE (Fig. 4A and B). The calibration curves showed good concordance between the predicted occurrence of PE by the model and the actual occurrence of PE in the training and validation cohorts (Fig. 5A and B). The DCA of the net benefit and cutoff probability (Fig. 6A and B) showed that the nomogram provided a favorable net benefit for patients with PE over an extensive range of cutoff probabilities in both the training and validation cohorts.

### 3.4. Model discrimination comparison

**Figure 7.**
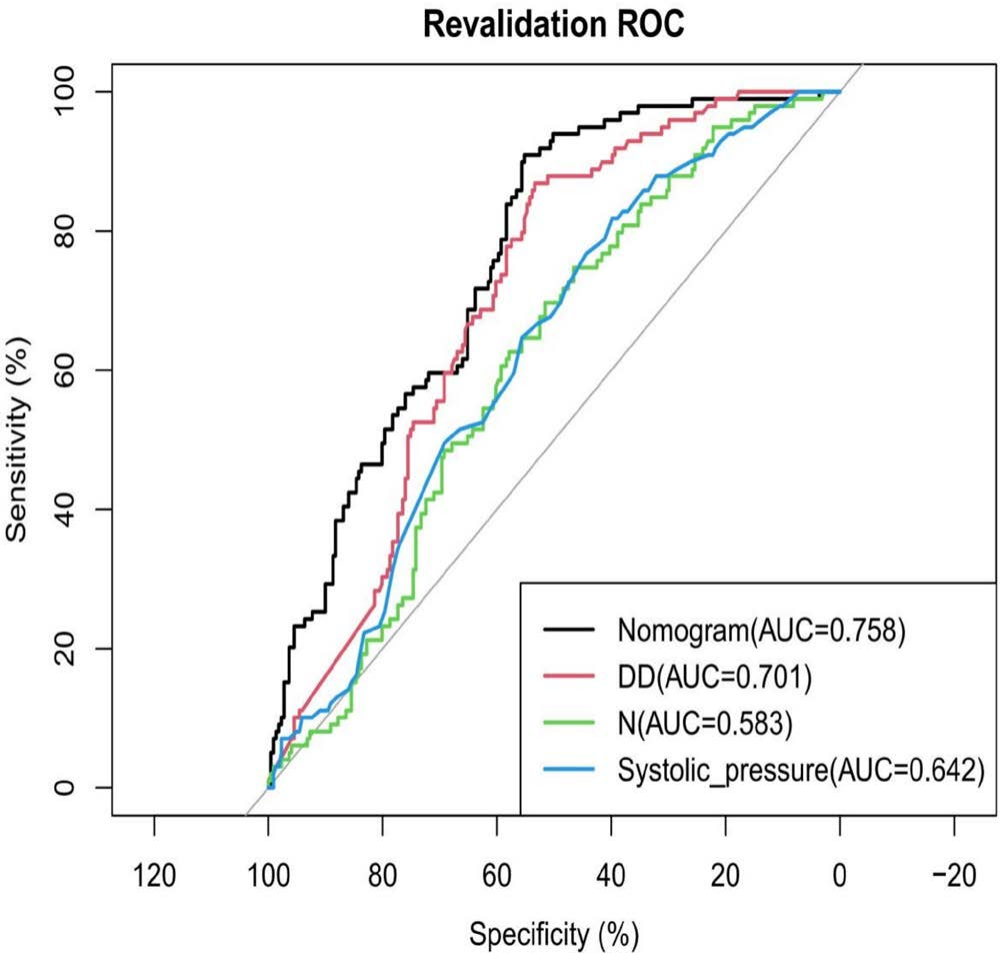
Model discrimination comparison. ROC curves of the nomogram (AUC-ROC is 0.758, black line), D-dimer (AUC-ROC is 0.701, red line), neutrophil count (AUC-ROC is 0.583, green line), and systolic blood pressure (AUC-ROC is 0.642, blue line). AUC = area under the ROC curve, DD = D-dimer, N = neutrophil count, ROC = receiver operating characteristic.

When comparing the discriminative ability of the models, the nomogram model demonstrated better accuracy in predicting clinical outcomes compared with individual measurements (neutrophil count, systolic blood pressure, and D-dimer level), as shown in Figure 7.

## 4. Discussion

This study developed a new numerical model to predict the risk of PE in oncology patients by incorporating 5 predictor variables: neutrophil count, sex, systolic blood pressure, surgical status, and D-dimer level. All parameters were readily available as clinical features and routine biomarkers. ROC analysis revealed an AUC of 0.758 (95% CI, 0.695–0.804), indicating good discrimination and calibration. The DCA curve showed that most threshold probabilities in this model had favorable net benefits.

Surgery is a significant risk factor for PE development.^[24–26]^ Despite the availability of effective prophylactic measures, the occurrence of postoperative PE remains a challenge. It is important to carefully assess each patient risk and tailor prophylactic measures accordingly.^[27–29]^ Additionally, close postoperative monitoring, prompt diagnosis, and treatment of PE are crucial to minimize morbidity and mortality.^[30]^ The present study also showed that surgical status was an important indicator in the prediction model and increased the risk of PE. D-dimer levels are currently the only biomarkers used routinely to predict PE^[31]^; however, at conventional positivity thresholds, this biomarker is unlikely to have sufficient specificity in oncology patients. We observed an association between elevated D-dimer levels and higher PE (OR 1.11; 95% CI 1.06 − 1.16). This result was consistent with that of a previous study,^[32]^ which showed that high D-dimer levels may be associated with PE development. In our model, systolic blood pressure was negatively associated with PE development. Low systolic blood pressure is associated with an increased risk of PE-related mortality, according to a previous study.^[33]^

The association between sex and PE risk is a topic of interest in medical research. Certain studies^[34]^ have suggested that there may be a relationship between PE and sex, with women being at a higher risk for PE than men. Several factors have been proposed to contribute to this difference, including hormonal changes, pregnancy, and the use of hormonal contraceptives.^[35]^ However, other studies have suggested that the relationship between sex and PE may not be significant and that other factors such as age, underlying medical conditions, and genetics may have a more significant impact on PE risk.^[6]^ However, we found that men were at a higher risk of PE than women. A possible explanation is that more men (60%) than women with tumors were included in this study. Inflammation is another factor believed to play a role in PE development. Studies^[36]^ have shown that individuals with underlying inflammatory conditions such as autoimmune diseases or infections have an increased risk of developing PE. Inflammation can cause changes in the blood vessels, leading to blood clot formation. Additionally, inflammation can impair the normal functioning of blood vessels, making them more prone to damage and blood clots. This study showed that neutrophil count was an important indicator in the prediction model and that an increase in this inflammatory indicator would increase the risk of PE. Another study^[37]^ also showed that inflammatory factors affect PE risk, which is consistent with the findings of this study. The patients in this study were all hospitalized in the tumor ward and were confirmed to have tumors, with almost all types of common tumors, including lung, breast, and colorectal cancers. We did not evaluate whether tumor type can be used as an indicator for predicting PE, as no similar relationship has been found in previous literature and clinical experience. Another reason is that tumor type is a factor variable in data analysis, and there are many tumor types that are not conducive to statistical analysis. Three possible explanations for the discrepancy are: i) indicators with > 20% missing values were excluded; ii) our clinical data did not include such indicators; and iii) similar indicators were not included in the model.

Our findings suggest that nomograms using clinical features and biomarkers for the individualized assessment of PE in oncologic patients may identify patients at high risk for PE. For example, a total score of 250 points for an oncology patient corresponds to a PE risk of approximately 14.7%. Additionally, using a nomogram to visualize the model’s output makes it easier for healthcare professionals to interpret and communicate the results to the patients. The high net clinical benefit demonstrated by the clinical decision curve analyses suggests that our model has the potential to improve patient outcomes and reduce healthcare costs. By accurately identifying high-risk patients, our model can reduce unnecessary diagnostic testing and treatment of low-risk patients, thereby optimizing resource utilization and reducing healthcare costs. The proposed numerical model may help clinicians classify oncology patients as likely or unlikely to develop PE. This may reduce the need for CTPA. This model can help identify high-risk patients, assess thrombosis, and implement active and individualized anticoagulation therapies.

This study had several limitations. First, 7 indicators with a missing rate ≥ 20% were excluded because of the retrospective nature of the study. Furthermore, the sample size was limited, and variables were inadequate; for example, the absence of certain common inflammatory indicators may have impacted the establishment of the model. Finally, the data were collected from a single center; thus, their external applications may be limited.

In conclusion, we developed a novel numerical model that can predict PE risk in patients with cancer. This model can assist in the appropriate selection of PE prevention strategies and also decrease unnecessary CTPA scans and their adverse effects. Further research is needed to validate the model in different clinical settings and to assess its impact on clinical decision-making and patient outcomes.

## Author contributions

**Conceptualization:** Qiu Liuyi, Chen Jianping, Ma Xu.

**Data curation:** Chen Tenggao, Lu Yifang, Li Wenchen.

**Formal analysis:** Chen Tenggao, Lu Yifang, Li Wenchen.

**Funding acquisition:** Chen Tenggao, Lu Yifang.

**Methodology:** Chen Jianping.

**Validation:** Qiu Liuyi, Chen Tenggao, Lu Yifang, Li Wenchen, Chen Jianping.

**Visualization:** Lu Yifang.

**Writing – original draft:** Qiu Liuyi, Ma Xu.

**Writing – review & editing:** Qiu Liuyi, Ma Xu.

## Supplementary Material


