# Supplementary figures and images for: Establishing a nomogram to predict the risk of pulmonary embolism in tumor wards: A retrospective study
Source: Medicine (Baltimore). 2025 Nov 21;104(47):e45737. doi: 10.1097/MD.0000000000045737 (PMC12643731; doi:10.1097/MD.0000000000045737)

**Supplemental Figure S1: Figure showing variables with < 20% missing data.**

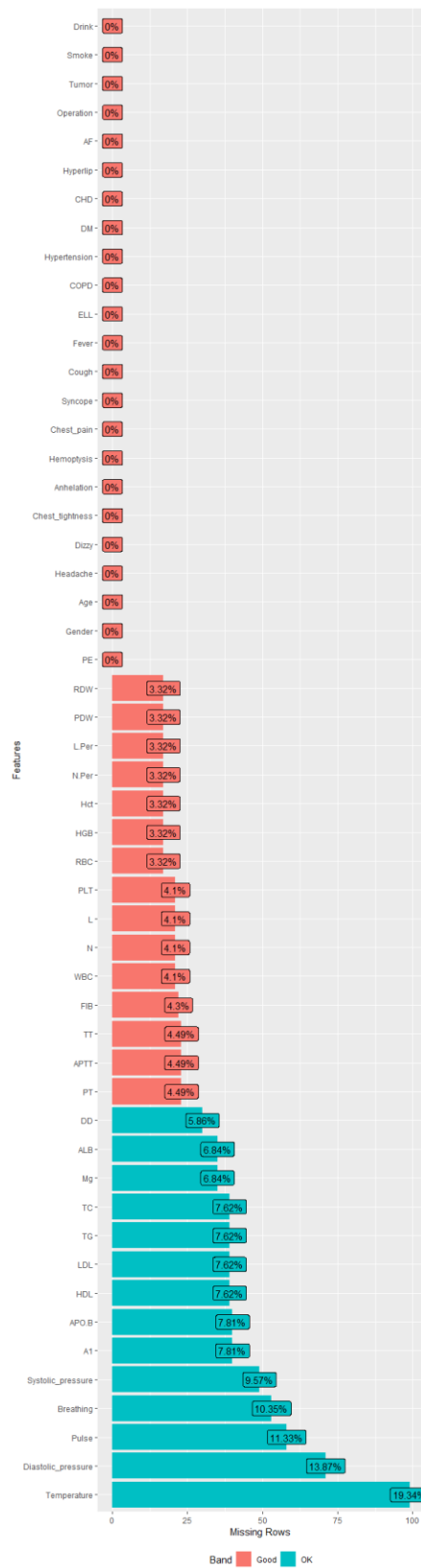

Supplement: Supplementary file 1 [file medi-104-e45737-s001.pdf]
